# What Patients Prioritize for Research to Improve Their Lives and How Their Priorities Get Dismissed again

**DOI:** 10.3390/ijerph19041927

**Published:** 2022-02-09

**Authors:** Barbara Groot, Annyk Haveman, Mireille Buree, Ruud van Zuijlen, Juliette van Zuijlen, Tineke Abma

**Affiliations:** 1 Department Public Health and Primary Care, Leiden University Medical Centre, Leiden University, Albinusdreef 2, 2333 ZA Leiden, The Netherlands; abma@leydenacademy.nl; 2 Leyden Academy on Vitality and Ageing, Rijnsburgerweg 10, 2333 AA Leiden, The Netherlands; 3 Centrum voor Cliëntervaringen, Jacob Bontiusplaats 9 (INIT-gebouw), 1018 LL Amsterdam, The Netherlands; annyk.haveman@uiteigenbeweging.nu (A.H.); barbarazzi@hotmail.com (M.B.); info@vanzuijlen.dds.nl (R.v.Z.); barbarazzi@gmail.com (J.v.Z.)

**Keywords:** patient perspective, epistemic injustice, community of practice, participatory health research, co-researchers, assistance dogs, assistive technology, abuse, dependency, peer support

## Abstract

Health researchers increasingly work with patients in a participatory fashion. Active patient involvement throughout the research process can provide epistemic justice to patients who have often only had an informant role in traditional health research. This study aims to conduct participatory research on patient experiences to create a solid research agenda with patients and discuss it with relevant stakeholders. We followed a participatory research design in 18 sub-studies, including interviews and group sessions (*n* = 404 patients), and dialogue sessions (*n* = 367 professionals and directors in healthcare and social work, municipality civil servants, and funding agencies) on patient experiences with psychiatric care, community care, daycare, public health, and social work. Findings from the eight-year study show that four priorities stood out: attention for misuse of power and abuse; meaningful participation; non-human assistance, and peer support. Moreover, that: (1) patients, based on their experiences, prioritize different topics than experts; (2) most topics are trans-diagnostic and point to the value of a cross-disability approach; and (3) the priorities of patients are all too easily dismissed and require ethics work to prevent epistemic injustice. Long-term investment in a transdisciplinary community of practice offers a solid basis for addressing patient-centered topics and may impact the quality of life of people living with chronic illness, disability, or vulnerability.

## 1. Introduction

There is increasing interest and support for the idea of patient and public involvement (PPI) in health services and, to a lesser extent, in health research. Examples are academics studying patient experiences and who identify as patients themselves [1,2,3,4] or who collaborate with people with lived experiences as their research partner or co-researcher [5,6,7,8,9,10]. More journals are also becoming patient-inclusive, such as the Patient Experience Journal with the Patients Included™ status. This status means that patients sit on the editorial board, routinely publish as authors, serve as peer reviewers, and provide open access. This all suggests a rising trend.

There are several arguments for including patients in research. Firstly, it is argued that patients possess unique experiential knowledge grounded in their lived experiences with the illness, vulnerability, or disability. This indicates that patients have singular perspectives on (coping with) their illness and treatment. This ‘emic’ or ‘insider’ perspective of patients is often as valuable and complementary to professionals’ ‘etic’ or ‘outsider’ perspective [11]. When patients are involved in research, this will enhance the societal impact and relevance. Secondly, it is argued that patients as end-users should have a voice in research that ultimately affects their lives. This normative argument is closely related to the ideal of epistemic justice—a concept coined by philosopher and feminist Miranda Fricker [12,13]. Epistemic justice is a commitment to acknowledge the fundamental human right to be respected as a bearer of knowledge. In line with this ideal, patients should be given a say in knowledge production.

Although the arguments for PPI are compelling, day-to-day practice shows that involvement is not a straight-forward and smooth process. Studies demonstrate that PPI’s implementation is highly uneven and that PPI is not yet firmly embedded or adequately formalized in European healthcare systems and research [14]. In practice, patients often do not have much influence or control over the research in which they are involved [15]. Patients, or clients who reflected on their experiences, are rarely engaged in a way in which their knowledge is valued equally and viewed as complementary in research, but are mostly involved at the levels of informing, consulting, and sometimes placation [16]. For example, patients are told about the study (informing), questioned in interviews or focus groups (consultation), and positioned on steering groups at specific moments in the research process (placation). In these settings, academics are still the initiators who determine the research agenda and control the research. Furthermore, patients living in vulnerable situations, that is, the ‘seldom heard’ [17], are rarely engaged [18].

The challenges of involving patients in research can be understood in the context of traditional power hierarchies in healthcare practices. Professional caregivers and health researchers have a privileged epistemic status based on expertise and thus risk (often unintentionally) downplaying patients’ testimonies and interpretations. Some are not viewed as credible knowers because of a negative identity and prejudicial stereotypes in healthcare [19], especially in psychiatry [20], but also in child and youth care [21], chronic illnesses like chronic fatigue syndrome [22], and chronic pain [23]. Patients’ testimonials can be disputed, for instance, because they do not follow the medical model [24]. Professionals may also (mis)judge patients’ intelligence, credibility, and rationality based on their language skills and discourse [25]. Epistemic injustice thus impacts the quality and equity of care provided and limits research on patient experiences.

Recently, frameworks have been developed for PPI and, more for specifically, patient-led priority or research agenda-setting [26]. In these types of studies different groups, including patients, are involved in drawing up an agenda of topics that are important to investigate. These studies value patients’ priorities as being of equal importance as the priorities of researchers and care professionals [27], and often focus on one diagnosis or patient group [28,29] or places and times defined by experts, like stays in a hospital [30]. Based on a systematic review of PPI frameworks, Greenhalgh and her team [26] suggest that researchers should develop and co-create a framework rather than choose one. How to co-create such a framework in a particular context, living up to the ideal of epistemic justice, and what kind of topics are prioritized when patients are genuinely involved in the agenda-setting from conception to conclusion, remains unclear.

The study presented in this article fills that void. It aims to create a solid patient-led research agenda amongst patients and discuss this agenda with relevant stakeholders (healthcare professionals, researchers, and funding agencies). We developed a framework together with stakeholders, including patients. Our findings show that (1) patients, based on their experiences, prioritize different topics than experts; (2) most topics are trans-diagnostic and point to the value of a cross-disability approach; (3) the priorities of patients are often too easily dismissed and require ethics work to prevent epistemic injustice.

When we use ‘patients’, we refer to people with chronic illnesses, psychiatric vulnerability, learning disabilities, or older adults currently receiving care. The term also refers those experiencing health problems due to poverty. ‘Experts by experience’ refers to patients who have reflected on their lived experiences, which are often life-changing. These experts often work as an advocate of the perspective of patients or use their skills and experiences to support peers and collaborate with professionals and researchers. We are aware that they may identify themselves as clients, users, end-users, service users, survivors, or people with an illness, vulnerability, disability, or living in poverty. In this article, we also use the term ‘co-researcher’ for anyone participating on equal footing with academics and who are often experts by experience.

## 2. Materials and Methods

### 2.1. Context: Learning Community on Patient-Led Care

In 2015, a small group of researchers and representatives of a patient advocacy organization with a shared mission to improve the quality of care for people living in vulnerable situations launched the Center of Client Experiences in the region of Amsterdam, The Netherlands (in Dutch: Centrum voor Cliëntervaringen, CvC for short; www.centrumvoorclientervaringen.com accessed on 5 February 2021). All members became ‘partners’ in the CvC learning community and strived for patient-led care, but felt alone in their mission. They often experienced resistance to their work that incorporated lived experiences or their collaboration with patients. The Center’s researchers sought partners who shared their mission and wanted to build a community to learn with and from each other about patient-led care.

Between 2014 and 2021, the CvC network grew to include ten organizations as official community partners and 19 patient co-researchers. The community partners met in face-to-face work sessions. The workshops’ agenda was decided by the group. We conducted sub-studies on patients’ experiences in various contexts to improve care with and for patients, together with partners. In parallel, we hope this will create more room and acknowledgment for the client’s perspective in care.

### 2.2. Methods and Analysis: Participatory Health Research

This article reports participatory health research (PHR) [31,32,33] in which 18 studies were conducted of lived experiences in psychiatric, community care, daycare, public health, and social work aimed to generate impact on the lives of patients (see Table 1). These 18 sub-studies drew on different groups of patients and co-researchers who all suffered from chronic illnesses or disabilities. In total, *n* = 404 patients shared their narratives in these sub-studies and *n* = 367 stakeholders (healthcare professionals, policymakers, funding agencies, municipal civil servants) were involved. The sub-studies’ aim was not to compile a patient agenda, but to foster change in practice. This research presents an analysis and reflection on the significant topics on the group’s agenda. In addition, the authors facilitated 12 three-hour work sessions at the CvC with 10–20 people each. The sub-studies and work sessions took place from 2014 to 2021.

The analysis was a collaborative and iterative process. In PHR, it is done with co-researchers and is an ongoing cyclical process of data generation and analysis over an extended period [31]. During this process, actions such as writing a research proposal also arise from the research, and are then reflected upon again. Important tenets of the analysis were: (1) that it was systematic; (2) that it occurred in collaborative dialogue and deliberation; (3) that findings were validated in the interim; and (4) that the process is replicable, as described below [56,57].

First, creative analysis methods were chosen in this study to analyze with co-researchers. This is common in participatory action research. Creative methods of analysis, such as the Critical Creative Hermeneutical Analysis (CCHA) [58], provided an opportunity to interpret experiences in an approachable way. We conducted this CCHA with co-researchers in each sub-study, in addition to thematic analysis [59] by the researcher(s). The CCHA and thematic analyses informed each other. As a result, extensive deliberation based on experiences (quotes from respondents) took place during all the sub-studies. Themes that emerged are described in the reports (see Table 1).

Secondly, a similar process occurred at a generic level, i.e., across all sub-studies. The first author was involved in all the studies discussed in this article. Over the past few years, she analyzed the reports thematically. The resulting themes were regularly discussed at work sessions at the Center for Client Experience. These were also the themes that were central to new research proposals written with co-researchers.

Finally, in November–December 2021, the first author thematically analyzed all reports, work session summaries, and draft research proposals and arrived at four themes, namely (1) attention to misuse of power and abuse; (2) meaningful participation; (3) alternative guidance or assistance; and (4) peer support. These themes were discussed with the co-authors (co-researchers) and validated in online meetings and telephone calls (due to the COVID-19 lockdown).

### 2.3. Author Team

The study’s author team is a mix of academics and co-researchers. The first and final authors are academic participatory action researchers who facilitated building the CvC and were involved in all 18 sub-studies as a researcher. The second author is a change agent with a mix of patient identities. The third author is an expert by experience in having a learning disability, the fourth with a chronic disease and physical disability, and the fifth with informal caregiving of her husband. The second to fifth authors were all involved in one or more sub-studies. Additionally, they were engaged in the CvC from the start.

## 3. Results

The results firstly describe the topics on patients’ research agenda for improving the healthcare system and policy (see Table 2). The shared topic of the agenda is ‘dependency.’ Patients are dependent on relatives, caregivers, organizations, procedures, and potential employers for (voluntary) jobs. Alternative support by peers, experts by experience, technology, and assistance dogs could support patients to become more independent. Patients’ dependency is a vulnerability, and misuse of power could lead to abuse by others.

The topics are not listed in a specific order; the first is not more important than the fourth. After the description of the research agenda, the findings from the dialogue with professionals on the agenda topics are shared.

### 3.1. Topics on the Patient Agenda

We describe four main topics of the patient agenda. These are the priorities of a broader list of issues. It is important to note that the topic of dealing with the restrictions, lockdowns, and other effects of the COVID-19 pandemic was critical at that moment. Still, we chose not to include pandemic topics in this patient agenda. More information on the priorities around COVID-19 from the patient perspectives of the CvC can be read in our report [49].

#### 3.1.1. Awareness for Misuse of Power and Abuse

In many sub-studies, participants shared with us experiences about misuse of power and situations of abuse (sexual, emotional, physical, or financial) in their daily lives or in their past. Their dependency of caregivers, family, partner or friends was often the core of the experience.

*“Our relationship? When it’s good, it’s very good. When it’s terrible, it’s very terrible. We can hurt each other a lot, but we also mean everything to each other. We will never forget that this is an abusive relationship, and like many abusive relationships, people fall away until at the end you are left with just the two of you.”*.

A couple who receives community care

*“Eventually, a group of 6–10 care professionals stood in the hallway to possibly jump on me. I found it threatening myself that they were all standing there. Then I ran away. Out of the door. The police went after me. I was taken away in handcuffs to the ambulance. Too bad. I found this very traumatic. I was crying. This is bad. I see that in the experiences of others too.”*.

A patient of emergency care

*“Care professionals ask at the start of counseling if sexuality should be a topic in counseling. If the checkmark is ‘off,’ it is never brought up again. However, this can change in practice. Situations and wishes can change. But they do not. If the checkmark is ‘off,’ the topic is never brought up again. Although things happened…”*.

A patient of community care

In all cases, the topic was not on our prepared interview guide. Still, during interviews about experiences in life concerning their health status, participants mentioned one or more forms of abuse (sexual, physical, or emotional), usually when the audio recorder was stopped. For example, co-researchers often introduced themselves as persons with patient experiences and difficult moments in life. In the interview, many participants referred to their own hard lives. They did not share the experiences in detail, but named the topic of abuse in passing. Introducing the co-researchers seemed to encourage people to relate similar experiences. Some participants felt free to share details about abuse in the interview; some did not.

In all sub-studies involving people with a learning disability, many participants shared experiences of childhood abuse, misuse by their partner, or even abuse at the daycare facilities or by formal caregivers. Most of the time, the abuse was linked to the perpetrators’ codependence. The perpetrators were often relatives, loved ones, or caregivers of people entirely dependent on them. However, most participants in this study did not expect to tell us about their experiences with abuse. We often heard that they had never told anybody in community care. They thus did not receive support for dealing with abuse and as a result, the incidents still impacted their health status significantly.

*“Nowadays, you can’t just trust people. That’s why I don’t have any friends really. I used to go through a lot with friends, so you think...”*.

A woman with a learning disability in an interview

In another study in the context of GPs, we found they were most frequently used by women, and almost all of them had experiences of abuse in their daily lives or in their past. The abuse was a critical topic for them, but also taboo. It seemed challenging to share the issue with formal caregivers. Participants did not feel comfortable discussing it, although most had weekly contact with the GP.

#### 3.1.2. Meaningful Participation

A second topic on the agenda is meaningful participation. In many sub-studies, patients share narratives about being a person who contributes to something or who cares for another. The topic of being seen, heard, and mattering emerged across almost all interviews. Many patients in the sub-studies cannot work in a paid or a voluntary job. Most organizations who provide a job are not as inclusive as patients wish and need. However, a job is an important way to build identity in Western society. A job ensures that you are seen (either positively or negatively), heard, and belong to a group or an organization, or enables you to break away from the patient role. A paid job can significantly improve quality of life and reduce dependence on a helping relationship. Besides, performing a task or helping someone, even if a small gesture, was a means to receive feedback and be part of a bigger whole.

*“When you are considered a vulnerable person, you have to work incredibly hard to be still able to do what you want to do. For example, if you’re in a wheelchair, they talk about you with the person who pushes the wheelchair. Then you don’t matter. So precisely when you’re vulnerable, you have to work harder to matter still. Because of the vulnerability.”*.

A person with a physical disability in community care

*“I worked at the X [an organization that supports homeless people]. That was the only place I could go for a job. But those are all homeless people. I just wanted to get out of that circle. I slept with addicts and drug dealers. I wanted to get away from that. So I’d rather not go back into that again.”*.

A woman with a psychiatric vulnerability

*“I could start there, but on an on-call basis. (...) That doesn’t really work for someone who needs a lot of structure.”*.

A man with a psychiatric vulnerability

For example, in the sub-studies on people with psychiatric vulnerabilities, we heard several experiences of people who no longer socialized and have become isolated and afraid of meeting others. Some participants sought to have a meaningful life by providing daily care for various family members. Informal caregiving was a significant job for patients. In the studies on informal caregiving, we found that most informal caregivers were also patients themselves.

*“I used to go to the snack bar a lot. But that all costs money. Sometimes I go to my mother’s house for dinner a few times a week. My mother has an eye disease. I will take her to the hospital then. And sometimes she needs help reading because of her sight. Then I do that. Then I read to her.”*.

A patient with a psychiatric vulnerability

In another study on experiences of community care for people with physical disabilities, patients shared stories about the role of caregivers in facilitating patients to participate in various activities. This included support for finding a (voluntary) job or hobby as well as with organizing the household and administration, so the patients had time to spend on social participation and volunteering. Many patients (would like to or did) managed their care to have energy left to spend on meaningful participation. A case illustrating this topic is a vivacious woman of 94, who spoke a lot about her activities and occupations. She has many social contacts, a good relationship with her son and daughter-in-law, and often goes out. For her, reciprocity is a significant value.

*“I have many friends who keep coming. I am very proud of that. Sometimes I say, if it’s too much trouble, you don’t have to. But they even say, I look forward to it. It is always so nice. (...) They come here for fun. Thank goodness. They are busy enough.”*.

Woman with community care

Her story shows that she can arrange her life, thanks to professional caregivers. A community caregiver supports her in her daily care, and (voluntary) organizations help her with transportation. Only in this way can she ease the burden on her family, monitor reciprocity, and participate in, what is for her, a meaningful way.

#### 3.1.3. Non-Human Assistance

Patients, especially people with a chronic illness, disability, or vulnerability, note that medications and therapies do not always solve problems, help them in meaningful participation, reduce pain, or support recovery.

*“Pills. Yes, I get those from the family doctor. But I don’t take them. I don’t want to. That’s junk. but yes, I’m still in that pain.”*.

A woman who frequently comes to the GP

In the sub-studies, we see that different people find support from animals like cats and assistance dogs, or smart assistive technology. This can be seen as non-human assistance. In the Netherlands, such support is not standard, as in the Dutch health system, assistance and support is mainly provided by humans, like physiotherapists, caregivers, physicians, social workers, etc. Non-human aid or support is often not evidence-based or accepted, and only a few healthcare organizations or foundations provide this type of care. It is thus difficult to arrange it without buying it yourself with private money.

To illustrate this topic, as an example, there is the fourth author, who has multiple sclerosis, cannot use his hands, and is dependent on a wheelchair. He found that devices with Bluetooth and WiFi connectivity can help him with many daily activities. In sub-studies, we also heard this from other patients:

*“I can have a job due to voice-recognition software connected with my smartphone on my wheelchair. I write emails, and call people with the Siri assistant. I can organize my life from my smartphone. This makes me less independent of care.”*.

Man with assistive technology and community care

However, in practice, the consumer devices that help patients with activities are not readily available at the municipality’s care desk or through the health insurance provider. Most (female) caregivers are not interested in technology and are even afraid of pressing a button on the wheelchair or smartphone. Finally, nobody feels that they ‘own’ the topic of assistive technology. Therefore, change in this area is not occurring yet. All patients who could benefit from smart assistive technology need to reinvent the wheel.

Another example is the second author, who has a psychiatric vulnerability, received regular psychiatry treatments for years, had several crisis admissions, and eventually found support from an assistance dog. As an assistance dog for people with sight impairment or other physical disabilities, the dog also helps prevent psychosis. The author’s dog is sensitive to her mood and trained to give signals that even she does not see. The dog becomes restless if she does not feel safe, so is a mirror for her well-being. In the sub-studies, we saw a valuable role for animals in support:

*“I still have one cat, and luckily, she is very stable now, and I am very grateful for that. The other two, I had to finish their lives. Both had a cancerous tumor. Finally, the tumors killed them. They were so involved and intertwined with me because I cared so much about them, and they were my best therapists who helped me out of a deep valley and kept me on track.”*.

A woman with a psychiatric vulnerability

However, this type of assistance is not a regular ‘product’ offered by the insurance provider or municipal support either. In different sub-studies with people with chronic psychiatric vulnerability, dogs are seen as a non-human form of assistance that is undervalued in the Dutch regular care system. These same experiences were shared in different studies, for example, where health professionals undervalue the impact of community garden projects, dancing projects, or yoga practices.

#### 3.1.4. Peer-Support

In almost all studies, patients suggested that peers could be of value for them. Peers share a similar experience and situation. This identity of a peer is under-researched in healthcare. In psychiatric care, peer workers are increasingly part of healthcare teams. However, peer support is still new and undervalued in many other settings, such as healthcare for adolescents, chronic illnesses, people with a learning disability, and in community care or informal caregiving.

In a study of adolescents with respiratory disease, the participants shared experiences with peers in group sessions. They found this very useful, learned from each other, and received a different type of support from their peers. In healthcare, all treatments and therapies are solo, although these participants would love to share their experiences in a group. This could help them even more, they thought.

*“You can tell your other friends, but they won’t understand. You feel supported, since you are not the only one.”*.

Adolescent with a respiratory disease

Patients in community care voiced similar feelings. The idea that people are eager to share their experiences in interviews with other co-researchers and love to join discussion groups is also a signal that peer support is valuable for them. According to patients, research on the value of peer support and the barriers of care professionals to be open to peer support is needed.

Peer support should not be confused with group therapy; it can also mean a group of people with a learning disability doing theater or a group of family caregivers engaging in a joint painting activity. It is not only about exchanging verbal experiences, but also about doing something together that allows the patient to meet people on a different level. Besides, peer support could inspire people or help them have hope for the future.

*“Experiential experts are currently being used more and more in psychiatry. However, these have different statuses and power. The exchange is no longer ‘really’ reciprocal, like with peers.”*.

The second author in an analysis work session

*“I had an interview with an elderly lady that the kids did everything for. She felt like she couldn’t do anything anymore. And then she saw me. A man in a wheelchair with many more limitations. And he comes independently by cab and does research. This inspired her enormously. She couldn’t imagine a situation where she would ever have such a role, but I gave her hope for the future.”*.

Fourth author in a work session

### 3.2. Reactions of Stakeholders on the Topics of the Agenda

Interestingly, some topics resonate more than others and were easier to follow up on.

#### 3.2.1. Attention for Misuse of Power and Abuse

Many care professionals in support of people with a psychiatric vulnerability and learning disability know that misuse of power by others is a topic in the lives of their patients. However, for general practitioners, community care, researchers, and municipal civil servants, different kinds of abuse are less familiar, often taboo, which creates a gap between theory and practice. Most professionals mention that the topic of abuse is far from their lifeworld. It was not prominent in their education, and the majority think it is only a topic for psychiatrists. However, patients indicate that it is a theme, although complex, that should be given a place in healthcare because they perceive that many physical and mental problems are related to experiences of abuse. Engaging in a dialogue about these themes proved a challenge in this study. The question raised by professionals was: who is responsible for this topic and how do you start? Their practical expertise on this topic is limited, and they expressed hesitance. It was often coined as a wicked problem like systemic racism. Therefore, according to patients, besides better education and information on abuse, additional research is needed from patients’ perspectives.

*“Sexual abuse is still related to rapists—man in bushes. Or nowadays, people think of #MeToo. Men who rape in a working relationship. However, most abuse comes from men in relational sphere. At home. In families. Many professionals do not know this or understand that their patients are one of those people who are a victim. They often do now know what to do to empower these women.”*.

Change agent who summarizes the discussion in a dialogue session

#### 3.2.2. Meaningful Participation

Since 2014, healthcare policy in the Netherlands has focused on participation, thus there were funding opportunities for participation in the period of this study. For care professionals and policymakers, the focus was on ‘more’ participation, regardless of the activity. For patients, however, emphasis was on ‘meaningful’ participation, which was defined as generating a sense of belonging or activities that contribute to society as a whole. Stability of a (voluntary job) was also as important for patients. Professionals and policymakers welcomed the insights from the patients’ perspective:

*“The titles of your reports ‘Not only participation works, but meaningful participation works’ and ‘Self-reliance, thanks to care’ make it clear to us that the principles of our policy work out differently in practice. This is an eye-opener for us.”*.

An municipality civil servant

#### 3.2.3. Non-Human Assistance

On the topic of ‘non-human assistance,’ some alternatives resonate with stakeholders more than others. For example, assistive technology seems to fit policy priorities and funding opportunities. As a team, we succeeded in funding one small project to study the experiences of people who could not use their hands with assistive technology. During this process, it became clear that, in practice, there is often insufficient knowledge among healthcare professionals to implement technology successfully. We also found a gap between what municipalities and health insurers offer and what is required in practice.

As far as our attempts to find funding for a study on assistance dogs and the value of animals in support, we found none. Although there is a growing literature on the value of assistance dogs and animals for health, care professionals were not eager to join us in this topic, and funders were not keen to finance this study proposal.

*“There are two types of assistance dogs: more task-oriented and more sensitive dogs. The task-oriented dogs are used by people, for example, with vision impairments and police and ambulance personnel. The more sensitive dogs are more focused on interdependence and working with people with trauma, veterans and/or psychotic symptoms. In uniform professions, people who work with these dogs increasingly receive financial assistance to purchase dogs. Unfortunately, this is not the case for patients with psychosis susceptibility or PTTS. However, dogs can play a vital role in reducing re-experiences of the traumas, panic attacks, and expensive compulsory treatment, and can prevent traumatic admissions. In addition, assistance dogs can provide a solution since the new law in the mental health sector, where patients are forcibly treated at home. Patients today are often administered their mandatory medication at home, where their own home is no longer considered a safe place. The assistance dog can help to experience and support safety. Unfortunately, psychiatry is still secondary to the care of people with visible disabilities or physical impairments. That’s also where the money for research is. That’s where the priority lies. Moreover, many do not see animals as complete: people can do it better, they think.”*.

One of the co-researchers after the unsuccessful process of funding a proposal on assistance dogs for patients with psychosis susceptibility and or other mental health issues

#### 3.2.4. Peer Support

Finally, the topic of ‘peer support’ is associated with the topic of meaningful participation. This study found that being an expert by experience could be a meaningful and recovery-promoting activity for clients. While the recommendations of our studies in 2014–2017 were at the time ‘new’ to stakeholders, since 2018, experts by experience have become a hot topic in social work. However, peer workers or the contribution of experts by experiences and research opportunities on experiences are still rare in hospitals, community care, at the GP, and in healthcare for adolescents.

*“Initiating an advisory board with patients is already a big deal in the department. But now, we also have to do something with the advice of these people. That is often quite difficult to achieve. So working together with experts by experience or peer support groups is a bridge too far.”*.

Physician in an academic hospital

*“The fact that we are researching patients’ experiences in psychiatric crises is already very innovative. If we do participatory research, I am not taken seriously. In the department here, hierarchy is still important and also the gold standard of medical research. It is challenging to fit participatory research in this system. Maybe in a few years...”*.

Emergency care professional in an academic hospital

Moreover, organizing peer support groups requires facilitation. In healthcare, most professionals say they lack training for this—it is considered a social work activity. What is needed here is interdisciplinary collaboration to organize peer support. Therefore, patients see the urgency to study this topic.

## 4. Discussion

This study created a solid research agenda of patients. Findings show that patients prioritize four topics that related to dependency: research on (1) awareness of potential misuse of power by others and abuse (sexual, emotional, physical, or financial) as a result; (2) support for meaningful participation of patients; (3) ways to implement non-human support and assistance; and (4) the implementation of peer support groups and peer workers in healthcare. Surprisingly, these topics were easily dismissed again in dialogue with stakeholders like health professionals, policymakers, and funding agencies. Below, we present the expert knowledge available of the priorities placed on the agenda by patients and how their priorities relate to and are handled by the academic community.

*Available knowledge on patients’ priorities, and an action-orientation that is missing.*

The priorities set by the patients in our studies are not (entirely) new for academics. For example, several review studies show the long-term health consequences of physical and emotional abuse, neglect, and sexual abuse [60,61,62], its prevalence [63], and disclosure [64]. However, research on economic abuse for adults in vulnerable situations is still a growing field [65]. Interestingly, most research on abuse had a low level of patient involvement. Furthermore, many of these studies recommend more randomized control trial studies (RCTs) and high-quality meta-studies to choose promising interventions for patients.

We see a similar pattern regarding ‘alternative support and assistance’. Here, we also identify a gap between the priorities of patients and the recommendations for further studies in reviews. Many reviews and qualitative studies show the benefits for health of assistive technology [66,67,68], assistive dogs [69,70,71,72], or companion animals in general [73,74,75,76,77], but recommend gaining more knowledge from quantitative research designs. Only a few studies propose a national policy on a topic, for example, assistive technology or the development of a framework for changing its implementation, e.g., [77].

We have shown that patients’ priorities are only partly covered in the literature, as scholars focus on more and better evidence assuming this will lead to a better quality of care by proven interventions. Patients are action-oriented and want academic research that stimulates tangible response to improve their quality of life. Patients also seek to learn what works in practice and co-produce new interventions that are relevant for their situation. Experts are therefore invited by patients to pay more attention to implementing knowledge to support patients in daily life.

*How the priorities set by patients was dismissed again.*

Many patient priorities, especially ‘the hard-to-reach,’ differ from the topics highlighted by professionals and researchers. This is often the case in agenda-setting studies [78,79,80,81]. Topics prioritized by patients represent their needs and wishes regarding scientific research. These issues reveal white spots that have been bypassed or superficially covered to new lenses on chronic illness and disability. While health science researchers tend to focus on highly specialized topics, patients focus on a broad array of interrelated issues of living with a chronic illness or disability. Moreover, researchers tend to focus on (para)medical treatments and psychological interventions, while patients also draw attention to the existential and social world of illness or disability [82]. In fact, patients constantly stress the importance of not being reduced to their patient role, but being seen as persons in their lifeworld context. As these issues are interrelated, researchers are invited to collaborate on the interface of humanistic and social scientific research with (bio)medical research. This requires transdisciplinary collaborations between academics, healthcare professionals and practitioners, and patients.

A systematic evaluation showed that one of the biggest challenges with patient-led research agenda-setting is that the topics generated may not be picked up by the funding agencies and researchers [83]. This was partly the case in our projects: some issues were well-received; others did not resonate with these stakeholders. In all instances, the facilitators teaming up with other partners in the community had to play a key role. They took responsibility for tying a certain topic to the scope of a funding agency. In the cases of assistive technology and meaningful participation, this was successful [54]. In the other instances, the facilitators looked for opportunities, but failed (thus far) to find resources to study the topics put on the agenda by patients. This leads to the conclusion that opening spaces for patient knowledge within academia is precarious and requires ‘ethics’ work to address power issues [5,84,85,86,87].

Finally, often patients are no longer involved in studies on the topics they prioritized [88]. In our case, the PPI and partnership had a more enduring status and resulted in a stable inclusion of patients in decision-making processes in a community of practice at the Center for Client Experiences (CvC) [84]. The partners were all willing to play a role as change agents toward a more inclusive healthcare and society. They were intensively involved and continued to act as advocates for the research agenda’s implementation and patient participation in health research. The CvC became a strong community; patients are well-informed, articulate, ambitious, and willing to participate in a study. These patients are strong players in terms of self-organization, self-awareness, and assertiveness, which are sometimes lacking in other instances where patients are not well integrated. The CvC is thus an ideal community with members capable of becoming and remaining equal partners in research.

## 5. Conclusions

Initiating involvement and partnerships between patients and parties with different scopes, interests, and research agendas can be challenging. The research agenda-setting presented showed that patients as part of a larger community can be connected in a process to begin an exchange and broaden perspectives on health research agendas. This led to a new set of research topics that mattered to patients and covered several domains of life, extending beyond specific diagnoses. Yet, these priorities were easily dismissed again. We thus recommend a cross-disability, participatory, and deliberative-dialogical approach as a framework for agenda-setting in the future to generate research priorities resulting from a deliberative-dialogic process facilitated by, and embedded in, a stable transdisciplinary community.

## Figures and Tables

**Table 1:** The background of the 18 sub-studies.

Field of Experience	Participants and Co-Researchers	Methods	Patients Involved	Stakeholders Involved	Year	Dissemination	Funder
Community care	Patients with chronic diseases, physical impairments and older adults	Interviews and a dialogue session with stakeholders about the findings	*n* = 32	*n* = 14	2014–2015	Report [34]	A municipality
	Older patients with community care		*n* = 29	*n* = 5	2016	Report [35]	Care organization
	All patients with community care		*n* = 85,	*n* = 79	2017	Report [36]	A municipality
	Informal caregivers		*n* = 49	*n* = 23	2016	Report [37]	
	Informal caregivers of patients with dementia		*n* = 19	*n* = 25	2016	Report [38]	Social work organization
	Patients with a learning disability		*n* = 20	*n* = 14	2015–2016	Report [39]	A municipality
			*n* = 20	*n* = 30	2017	Report [40]	
			*n* = 15	*n* = 8	2019	Report [41]	
	Patients with a psychiatric vulnerability		*n* = 5	*n* = 17	2015	Report [42]	
	Patients who are dependent on assistive technology		*n* = 6	*n* = 4	2018–2020	Video [43]	Charitable foundation
General practicians (GPs)	Frequent users of GPs		*n* = 17	*n* = 5	2019	Web text [44]	Health insurance company
Emergency psychiatric care	Patients who want to be admitted voluntarily		*n* = 17	*n* = 32	2019	Report [45]	Health insurance company
	Patients in psychiatric crisis		*n* = 17	*n* = 28	2017–2018	Report and articles [46,47]	Two psychiatric care institutions
Social work and public health	People ageing at home		*n* = 40	*n* = 14	2016–2020	Report [48]	A municipality & Applied University
	Patients in COVID-19 isolation	(Online) Group sessions with session with stakeholders about the findings	*n* = 9	*n* = 21	2020	Report [49]	Dutch Health Research Fund
	People living in poverty		*n* = 6	*n* = 25	2018	Report and articles [50,51,52]	Charitable foundation
	People without a job		*n* = 10	*n* = 19	2018–2019	Report and article [53,54]	A municipality
Hospital care	Youngsters with a respiratory disease		*n* = 8	*n* = 4	2018–2020	Article [55]	Dutch Foundation for Asthma Prevention

**Table 2:** The research agenda of patients based on 18 sub-studies.

Research Agenda of Patients	Explanation
Misuse of power and abuse	The misuse of power of (informal) caregivers and relatives on patients, with abuse (sexual, emotional, physical, financial) as a result
Meaningful participation	Support for patients to participate in a way that they are seen, heard, and belong to a bigger whole
Non-human assistance	Ways to implement alternatives support like assistance dogs and smart assistive technology
Peer support	Implementation on peer support and peer workers in settings like adolescent care, care for people with chronic illnesses or a learning disability, community care, and support for informal caregivers
